# Chitosan Scaffolds Containing Calcium Phosphate Salts and rhBMP-2: *In Vitro* and *In Vivo* Testing for Bone Tissue Regeneration

**DOI:** 10.1371/journal.pone.0087149

**Published:** 2014-02-04

**Authors:** Rodrigo Guzmán, Stefania Nardecchia, María C. Gutiérrez, María Luisa Ferrer, Viviana Ramos, Francisco del Monte, Ander Abarrategi, José Luis López-Lacomba

**Affiliations:** 1 Instituto de Estudios Biofuncionales, Universidad Complutense de Madrid, Madrid, Spain; 2 Instituto de Ciencia de Materiales de Madrid-ICMM, Consejo Superior de Investigaciones Científicas-CSIC, Campus de Cantoblanco, Madrid, Spain; 3 Noricum Inc, Departamento de I+D, Parque Científico de Madrid, Tres Cantos, Madrid, Spain; Institute for Frontier Medical Sciences, Kyoto University, Japan

## Abstract

Numerous strategies that are currently used to regenerate bone depend on employing biocompatible materials exhibiting a scaffold structure. These scaffolds can be manufactured containing particular active compounds, such as hydroxyapatite precursors and/or different growth factors to enhance bone regeneration process. Herein, we have immobilized calcium phosphate salts (CPS) and bone morphogenetic protein 2 (BMP-2) – combined or alone – into chitosan scaffolds using ISISA process. We have analyzed whether the immobilized bone morphogenetic protein preserved its osteoinductive capability after manufacturing process as well as BMP-2 *in vitro* release kinetic. We have also studied both the *in vitro* and *in vivo* biocompatibility of the resulting scaffolds using a rabbit model. Results indicated that rhBMP-2 remained active in the scaffolds after the manufacturing process and that its release kinetic was different depending on the presence of CPS. *In vitro* and *in vivo* findings showed that cells grew more in scaffolds with both CPS and rhBMP-2 and that these scaffolds induced more bone formation in rabbit tibia. Thus chitosan scaffolds containing both CPS and rhBMP-2 were more osteoinductive than their counterparts alone indicating that could be useful for bone regeneration purposes, such as some applications in dentistry.

## Introduction

One of the main topics in bone tissue engineering is the quest for functional materials capable of promoting tissue regeneration. Considering that bone is mostly composed of collagen and hydroxyapatite (HAp), biomaterials containing chemical-analogues and/or chemical-inducers of any of these compounds – or, ideally, of both – have been widely explored in the field of bone tissue engineering as they can eventually accelerate bone regeneration [1]. Thus, a wide number of mineralized and biomineralized scaffolds – with either HAp or other calcium phosphate salts – have been proved to be more efficient for regeneration purposes than the non-mineralized counterparts [2]–[4]. Interestingly, the use of low and non-crystalline forms of calcium phosphate salts (e.g. amorphous calcium phosphate, ACP) have been the preferred choice because the mechanism for HAp formation (i.e. based on dissolution and recrystallization) is favoured in presence of low- and non-crystalline salts as compared to more crystalline ones [5], [6].

Besides HAp precursors, a group of growth factors known as bone morphogenetic proteins (BMPs) has been extensively studied for applications related to bone regeneration. Bone healing processes involve a complex integration of cells, growth factors, and the extracellular matrix, and it is widely accepted that naturally occurring BMPs are critical players in these processes. In particular, BMPs are expressed in the early stages of fracture repair where it is likely that small amounts are released from the extracellular matrix of the fractured bone. Tissue that first bridges the fracture site is known as the fracture callus. Bone healing continues upon replacement of fracture callus with lamellar bone – typically in the form of trabecular bone – by a combination of endochondral ossification and bone mineralization processes – in charge of, respectively, cartilage and bone replacement. Healing eventually terminates upon a remodelling process, where part of the trabecular bone becomes compact bone and the excess of trabecular bone is resorbed by osteoclasts. The current view of the role of BMPs in described fracture repair process is that these molecules are primarily activators of differentiation in osteoprogenitor and mesenchymal cells destined to become osteoblasts and chrondrocytes which form woven bone and cartilage [7].

Therefore BMPs are osteoinductive and have been shown to increase bone formation in research studies [8]–[10]. Genetic recombinant techniques have made possible the production of recombinant human BMP-2 (rhBMP-2), the local administration of which have also induced ectopic bone formation, improved the healing of fractures and bone defects in animals and even stimulated bone formation by human bone marrow-derived stromal cells *in vivo* [11]–[14]. However, the rhBMP-2 performance upon *in vivo* administration may be limited by its short biological half-life and its rapid clearance after injection [15]. Hence, incorporation of rhBMP-2 into some biomaterials may improve its clinical therapeutic effectiveness. Moreover, rhBMP-2 release from these biomaterials may allow maintaining an effective local concentration, achieving prolonged availability, and preventing the systematic risk of high dose [16], [17].

Numerous strategies that are currently used to engineer tissues depend on employing biocompatible and/or biodegradable materials exhibiting a scaffold structure. On one hand, the structural similarities of these scaffolds with synthetic extra cellular matrix (ECM) are helpful for cell adhesion and serve to organize cells into a three-dimensional architecture. Additionally, scaffolds act as substrates where any of the above-mentioned compounds that induce bone formation can be immobilized and subsequently released. Herein, we describe the preparation of chitosan (CHI) scaffolds containing rhBMP-2, either alone or besides calcium phosphate salts (CPS). CHI is a cationic copolymer of N-acetylglucosamine and glucosamine of natural origin widely used in tissue engineering [18]–[22]. The preparation process consisted of an enzymatically-induced gelation of CHI (based on the urease-assisted hydrolysis of urea at room temperature) [23] followed by a unidirectional freeze-drying process [24]–[26] (also called ice segregation induced self-assembly, ISISA) [27], [28] that allows the formation of macroporous scaffolds with an excellent control of the structural features and a homogeneous distribution of any compound eventually immobilized within the scaffolding structure. We have recently described this process for the preparation of CHI scaffolds containing ACP (both alone and in combination with anhydrous ciprofloxacin crystals, a synthetic antimicrobial fluoroquinolone) [29], [30] but the immobilization of active rhBMP-2 – rather than the simple post-synthetic adsorption onto the scaffold [31] – requires some attention. In this case, the mild processing shows promising conditions for the activity preservation of the immobilized protein. With regard to the structural features of the resulting materials, we have used scanning electron microscopy (SEM) to study the morphology of the macroporous scaffolds. The mechanical properties as well as the swelling of the different scaffolds were also evaluated. With regard to the functional features provided to the resulting materials, here we have demonstrated that the intrinsic osteoinductive capability of rhBMP-2 was preserved when immobilized within the scaffold structure and we have also studied its kinetic release. We have studied the *in vitro* biocompatibility of the resulting scaffolds. Finally, we have created defects of 4 mm in diameter in rabbit tibias and evaluated the *in vivo* osteoinductive response of the scaffolds.

## Materials and Methods

### 1. Ethics statement

All animal handling and experimental procedures were approved by the Animal Care and Use Committee of Universidad Complutense, according to the guidelines for ethical care of experimental animals of the European Community.

### 2. Materials

Chitosan (CHI, Batch#06513AE, Av. Mol. Wt. 617 kDa, deacetylation degree (DD) 88±2), hydroxyapatite (HAp, Batch#07424MD, reagent grade), urease (from *Canavalia ensiformis* (Jack Bean) Type III, Sigma lot 115K7030, 45 units/mL) and urea were from Sigma-Aldrich and used as received unless otherwise indicated. rhBMP-2 (from *Escherichia coli*) was kindly supplied by Noricum S.L. (Spain).

### 3. Samples Preparation

#### 3.1. Urease-assisted preparation of hydrogels

Bare CHI hydrogels were prepared as described elsewhere. Briefly, an aqueous solution of CHI (1 g, 2.83 wt.% in acetic acid 0.15 M; pH 4.5) was mixed with an aqueous solution of urea (0.125 mL, urea 2 M, HCl 0.3 M; pH 3) under vigorous stirring in an ice-cold bath. The resulting solution was mixed with 0.0875 mL of a freshly prepared urease aqueous solution (45 units/mL) and stirred for 15 min. The resulting translucent liquid mixture was loaded into an insulin syringe (1 mL) and aged for gelation (24 hours at 37°C up to reach a pH of ca. 7.0).

Hydrogels containing rhBMP-2 were prepared following the procedure described above for CHI hydrogels except for the addition of an aqueous solution of rhBMP-2 in acetic acid 0.15 M (0.125 mL, 2 mg/mL) to the aqueous solution of CHI (1 g, 2.83 wt.% in acetic acid 0.15 M; pH 4.5). The subsequent addition of urea and urease was accomplished as described above. The resulting translucent liquid mixture was loaded into an insulin syringe (1 mL) and aged for gelation (24 hours at 37°C up to reach a pH of ca. 7.0).

Hydrogels containing CPS either without or with rhBMP-2 were prepared following the procedure described above for CHI and rhBMP2-CHI hydrogels, respectively, except for the addition of calcium phosphate to the urea solution (e.g. 0.125 mL, urea 2 M, Ca5(PO4)3OH 0.03 M, HCl 0.3 M; pH 3.0). The resulting translucent liquid mixture was loaded into an insulin syringe (1 mL) and aged for gelation (24 hours at 37°C up to reach a pH of ca. 7.0). All the solutions were previously sterilized by filtration (0.22 µm) and after the freeze-drying step they were handled in sterile conditions.

#### 3.2. ISISA processing of hydrogels

Freshly gelled hydrogels (CHI, CPS-CHI, rhBMP2-CHI, and rhBMP2-CPS-CHI hydrogels) were unidirectionally frozen by dipping the insulin syringes (at a constant rate of 2.7 mm/min) into a liquid nitrogen bath at 77 K. The frozen samples were freeze-dried using a ThermoSavant Micromodulyo freeze-drier. The process preserved the homogeneous distribution of every component throughout the entire volume of the resulting monolith. The resulting CHI, CPS-CHI, rhBMP2-CHI, and rhBMP2-CPS-CHI monoliths kept both the shape and the size of the insulin syringes (ca. 63 mm in height and 4.5 mm in diameter). The bottom part of the monolith was always disregarded because of structural heterogeneities [27]. For the *in vitro* and *in vivo* assays monoliths were cryo-fractured (i.e. prior the freeze-drying step) into scaffolds of 2.5 mm in height and 4.5 mm in diameter. The rhBMP-2 content in rhBMP2-CHI, and rhBMP2-CPS-CHI scaffolds was ca. 8 µg per scaffold.

### 4. Samples characterization

Sample morphologies were investigated by scanning electron microscopy (SEM, Zeiss DSM-950 instrument). The nature of CPS was studied by transmission electron microscopy (TEM, 200-KeV JEOL 2000 FXII microscope) as described elsewhere [30]. Swelling experiments were carried out at 20°C by immersing the scaffolds in an excess amount of deionized water. The swelling ratio was calculated as (W_2_−W_1_)/(W_1_) %, where W_1_ and W_2_ were the weight of the polymer before and after swelling, respectively.

Mechanical properties of the monoliths were evaluated by measuring tensile strength versus elongation. For this purpose, dry cylindrical monoliths of 20 mm in height (obtained by cryo-fracture, as described above) were gently pressed to obtain rectangular parallelepipeds of 20 mm in height, 4.6 mm in width and 200 µm thick. The strain rate was set to 1 mm/min. All experiments were conducted by triplicate.

### 5. *In vitro* assays

#### 5.1. Cell line

Cellular assays were performed using C2C12 cell line (pre-myoblast, mouse, CRL 1772) obtained from the American Type Culture Collection (Manassas, Virginia). Routine passaging of the cell line was performed on flasks with Dulbecco's modified Eagle's medium high in glucose, containing 10% fetal bovine serum plus antibiotics (100 U/mL penicillin and 100 mg/mL streptomycin sulfate) and maintained in an atmosphere with 5% CO_2_ at 37°C.

#### 5.2. Experimental design

Scaffolds (4.5 mm in diameter and 2.5 mm in height) were placed into a 48-well plate and seeded by triplicate with 10 µL of complete medium containing 2×10^5^ trypsinized cells per scaffold dropping-wise directly onto them. Plates were incubated at 37°C in a humidified 5% CO_2_ atmosphere for 45 min to allow cells be attached on the scaffolds. Afterwards, 400 mL of pre-warmed complete culture medium was carefully added, and the plate was maintained at 37°C in a humidified 5% CO_2_ atmosphere. Before any measurement and to avoid any contamination eventually coming from cells adhered onto the plastic surface of the well, every scaffold was transferred to a new well and fresh complete culture medium was added. Cultures were performed over three time points (24, 72 and 120 hours). Non-seeded scaffolds (i.e. without cells) placed in the same plate were used as blanks.

#### 5.3. Cell proliferation and viability assay

Alamar Blue assay (Invitrogen) was used to test cell proliferation at each time point, following manufacturer instructions. For this purpose, Alamar Blue reagent was added to culture medium (40 µl of reagent in 400 µl of medium). After the incubation period (37°C, 90 minutes), the medium was transferred to new wells and the fluorescence emitted at 590 nm - using λ_ex_ = 530 nm - was recorded after subtraction of blank readouts (Biotek FL-600). Final data were referred to those obtained at 24 hours in order to provide the relative growth after this time point.

Cell viability was also tested at every time point using the calcein AM assay (Molecular Probes, Eugene, Oregon, USA) and following manufacturer instructions. Briefly, cell culture medium was replaced with 400 µL of PBS-calcein AM (1 µg/mL). Cells were then incubated over 15 minutes at 37°C. Fluorescent images were obtained in an Olympus BX51 microscope (Olympus).

#### 5.4. rhBMP-2 activity test

The protein activity was evaluated by the ability of BMP-2 to induce alkaline phosphatase in C2C12 cells. Briefly, rhBMP2-CHI and rhBMP2-CPS-CHI scaffolds were dissolved in 100 µL of 50 mM acetic acid at 37°C over 1 hour. Then 10^4^ C2C12 cells were seeded in a 48-well plate using 400 µL of culture medium and 30 µL of every scaffold solution per well. Considering that the average rhBMP-2 content per scaffold was 8 µg, 30 µL of solution contained approximately 2.5 µg of protein. Thus, this was the amount of rhBMP-2 used as positive control, which was also dissolved in acetic acid 50 mM. The solution of CHI scaffold without protein was used as the negative control. Cells were incubated for 4 days, then washed with 200 µL of PBS and finally treated with 100 µL of lysis buffer (50 mM Tris pH 6.8, 0.1% Triton X-100, 2 mM MgCl_2_) per well. After three freeze/thaw cycles, 10 µL of samples were assayed for alkaline phosphatase activity in 96-well plates, using p-nitrophenyl phosphate in 2-amino-2-methyl-1-propanol buffer as the substrate and a total volume of 100 µL. After 10 min at 37°C, the reaction was stopped with 100 µL of 0.5 M NaOH and the absorbance at 405 nm was measured in a Microplate Reader (Biotek FL-600).

#### 5.5. rhBMP-2 release assays

The protein release from rhBMP2-CHI and rhBMP2-CPS-CHI scaffolds were analyzed at eight different time points (0.5, 1, 2, 3, 4, 5, 120, 216 hours) using specific sandwich ELISA assay (Peprotech, UK) and following manufacturer instructions. Briefly, a 96-well microplate was precoated with a rabbit anti-BMP-2 antibody overnight and blocked over 1 hour with 1% BSA in PBS 1X pH 7.4. Afterwards, 100 µL of culture medium was placed into the microplate and incubated over 2 hours. Then, biotinylated rabbit anti-BMP-2 antibody was added and incubated over an additional hour. After that, an Avidin-HRP conjugated was added to the microplate and incubated over 30 minutes. Finally, ABTS (Sigma) was added and the absorbance at 405 nm was measured using a Biotek FL-600 microplate reader. All the process was carried out at room temperature using the absorbance of a culture medium non-exposed to scaffolds as blank readout. Data obtained were converted to ng/mL by interpolation on standard curve. Total rhBMP-2 content was determined multiplying by culture medium total volume (400 µL).

### 6. *In vivo* assays

#### 6.1. Surgery

The *in vivo* studies were performed in New Zealand male rabbits of ca. 3 kg in weight. Ten rabbits were used with different replicates for each sub-group of implant (empty control, n = 3; CHI, n = 3; rhBMP2-CHI, n = 4; CPS-CHI, n = 4; rhBMP2-CPS-CHI, n = 6). The rabbits were anesthetized by intramuscular injection with 2% Rompun (1 mL/10 kg, Bayer) and Imalgene 1000 (ketamine 20 mg/kg, Merial) making all efforts to minimize animal suffering. The surgical field (tibiae maesetae of both legs) was shaved and disinfected. Defects of 4 mm in diameter were drilled and the scaffolds were implanted. Empty tibia defects were used as control. The rabbits were placed in individual cages with food and drink fully available. After a period of 3 weeks of implantation, the experimental subjects were sacrificed and samples were collected in formalin for further analysis.

#### 6.2. Micro-Computed Tomography

Samples were imaged with an X-ray tube voltage of 50 kV and a current of 200 µA. The scanning angular rotation was 180°, the angular increment 0.40°, and the voxel resolution 50 µm. Data sets were reconstructed and segmented into binary images (8-bit BMP images) for the subsequent image processing reconstructions using MicroView ABA 2.2 software (GE Healthcare).

#### 6.3. Histology and histomorphometry

Formalin-fixed samples were decalcified with 10% and 5% nitric acid over 3 and 2 days, respectively. Afterwards, samples were embedded in paraffin and the resulting paraffin blocks were cut in 4-mm-thick slides. The slides were subjected to hematoxylin/eosin and Masson's trichrome staining protocols and observed with an Olympus BX51 microscope. Histomorphometry was carried out on the slides stained with Masson's trichrome by measuring newly formed bone with the CellD software from Olympus. All the histological processing was performed by Dominion Pharmakine services.

### 7. Statistics

Every experiment was performed by triplicate. Data were expressed as means ± standard deviation (SD). ANOVA analysis was performed using SPSS software (SPSS Inc.) to compare mechanical properties values between different types of scaffolds. Furthermore, ANOVA was also used in proliferation assays – carried out to compare cell proliferation rates between scaffolds – and histomorphometric studies, using Bonferroni post-test to compare the different scaffolds with the control (empty defect). Statistical differences were considered significant when P<0.05. Non-linear regression fitting for rhBMP-2 release assays was performed with Graph Pad Prism software.

## Results and Discussion

**Figure 1:**
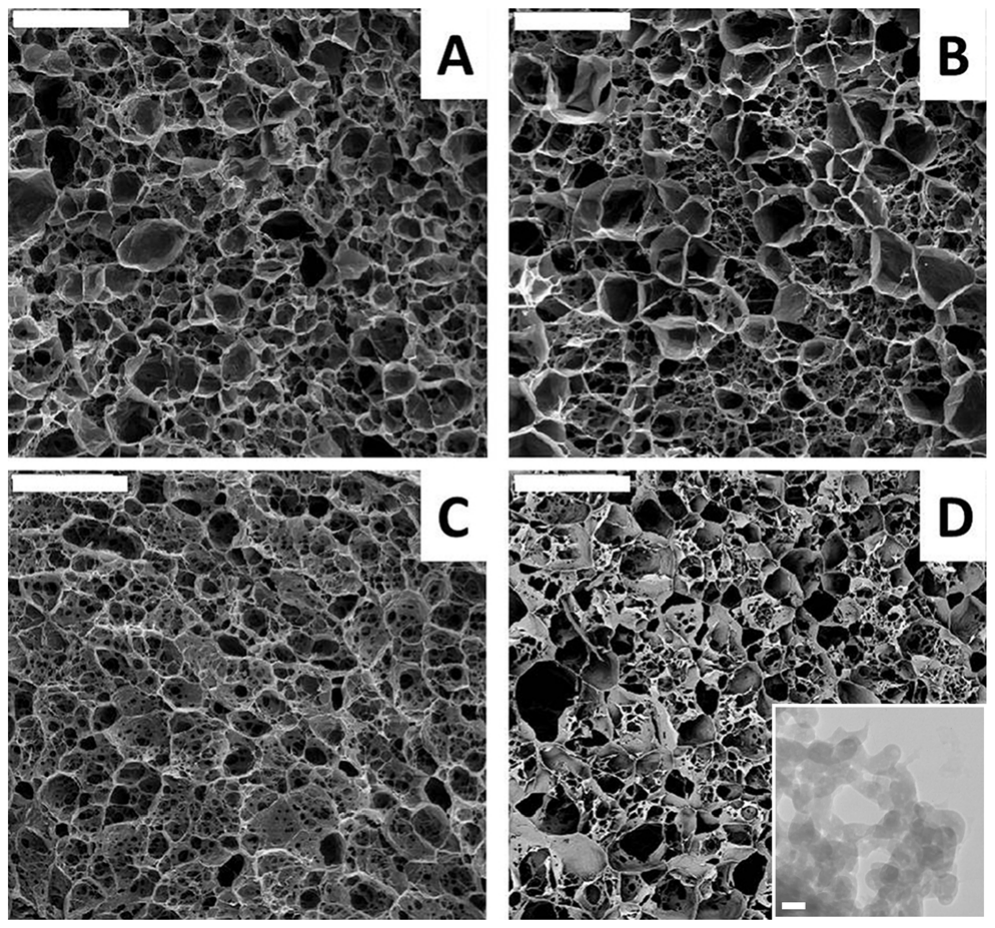
SEM micrographs. Images show the macroporous structure of CHI (A), CPS-CHI (B), rhBMP2-CHI (C), and rhBMP2-CPS-CHI (D) scaffolds. Bars are 50 µm. rhBMP2-CHI (C), and rhBMP2-CPS-CHI (D) scaffolds. Bars are 50 µm. Inset shows CPS in form of ACP in both CPS-CHI and rhBMP2-CPS-CHI scaffolds. Bar is 30 nm.

**Figure 2:**
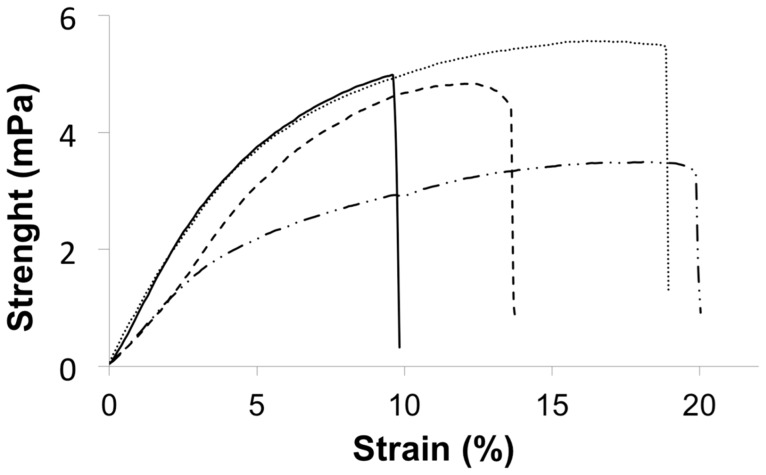
Mechanical characterization. Plots representing the tensile strength versus strain for CHI (—), CPS-CHI (**– –**), rhBMP2-CHI (**···**), and rhBMP2-CPS-CHI (**–··–**) scaffolds.

Our first interest was to study the morphology of the scaffolds resulting after the application of the ISISA process to the different hydrogels. ISISA is just an ice templating process so that upon freezing, the ice formation (hexagonal form) causes the hydrogel network (originally occupying the entire hydrogel volume) become segregated and concentrated between adjacent ice crystals. Freeze-drying resulted in the formation of structures consisting of micrometer-sized pores corresponding to the empty areas where ice crystals originally resided. Thus, ISISA is just like any other freeze-casting process. The only difference is that freezing is carried out by immersion of the samples into a cryogenic liquid (e.g. liquid nitrogen) at a constant dipping rate. This controlled-immersion process is critical to obtain a homogeneous macroporous structure throughout the entire monolith except at the zone of the probe that first comes into contact with the liquid nitrogen. In this area the material is dense because the temperature is so low that amorphous rather than crystalline ice is formed (i.e., water can supercool when either a solution or hydrogel is submitted to −196°C because of the presence of solutes) and matter segregation does not occur. After this first contact, an ice front appears running upward the non-immersed portion of the probe, the temperature of which – always above −196°C – will depend on the ice front height above the liquid nitrogen level; i.e. the higher the location of the ice front, the higher its temperature. As mentioned above, macroporosity corresponds to the empty areas where ice crystals originally resided so that the dimensions of the pores are ultimately governed by the dipping rate; i.e. large pores are obtained at slow dipping rates because of the favoured growth of ice crystals when the ice front is far from the immersion level. In our case, we always used an immersion rate of 2.7 mm/min. It is worth mentioning that we always disregarded the dense portion of the monolith (e.g. first 3–4 mm of the bottom part of the monolith) so that the cross-section images shown in figure 1 just revealed the macroporous structure of CHI, CPS-CHI, rhBMP2-CHI, and rhBMP2-CPS-CHI scaffolds. SEM micrographs revealed no remarkable differences between them as correspond to scaffolds with a common main component (e.g. CHI; see Figure 1). TEM micrograph allowed determining the major presence of CPS in form of ACP, that is, as “Posner clusters” of approximately 1.0 nm in diameter, which aggregate randomly forming large spherical particles of 20–300 nm with tightly bound water residing within the interstices (see inset in Figure 1) [30]. The swelling capability in phosphate buffer (PBS, 0.1 M; pH 7.4) decreased from CHI to rhBMP2-CHI to rhBMP2-CPS-CHI down to CPS-CHI (Table 1). The presence of CPS and rhBMP-2 also played a role in the mechanical properties of the scaffolds (Figure 2, Table 1). For instance, the elongation at break-point of CPS-CHI exhibited a significant increase as compared to that of its respective counterpart (e.g. CHI). A similar trend was observed for rhBMP2-CPS-CHI and rhBMP2-CHI, with a significant increase of the elongation at break-point in the scaffolds containing CPS. Meanwhile, the presence of rhBMP-2 contributed to the elastic character of the scaffolds with a significant decrease of the Young Modulus as compared to their respective counterparts. Interestingly, the elongation at break-point also increased in the scaffolds containing rhBMP-2 (e.g. 13.6% for rhBMP2-CHI versus 10.0% for CHI, and 21.4% for rhBMP2-CPS-CHI versus 18.0% for CPS-CHI). The improvement of the elastic properties of samples containing rhBMP-2 made easier their handling during surgery. It is worth noting that, from a practical point of view, this was by no means a trivial issue. This is in agreement with the behavior described in previous works for incorporation of charged species in polyelectrolyte hydrogels [32]. Thus, opposite charges results in attractive forces so that charged species ultimately work as cross-linkers – with the subsequent increase of the Young modulus – while when both the polyelectrolyte and the incorporated species have similar charges, the additive works as a plasticizer and the Young modulus decreases. This is actually our case, considering that CHI is a polycation and the rhBMP-2 charge at the pH where the hydrogel is formed (ca. 7.0) is slightly positive.

**Table 1:** Rheological properties (Young modulus, tensile strength, and elongation at rupture) and swelling ratio of CHI, CPS-CHI, rhBMP2-CHI, and rhBMP2-CPS-CHI scaffolds.

Scaffold	Young modulus (GPa)	Tensile Strength (MPa)	Strain (%)	Swelling ratio (%)
CHI	0.12±0.02	4.9±0.1	10.0±1.0^c^	1560
CPS-CHI	0.12±0.01	5.5±0.8	18.0±0.8^c^	902
rhBMP2-CHI	0.07±0.02^a^	4.8±0.5	13.6±0.7^c^	1250
rhBMP2-CPS-CHI	0.05±0.03^a^	3.3±0.9^b^	21.4±0.9^c^	1145

Data are presented as means ± SD.

a Significant differences (P<0.05) compared to the scaffolds without rhBMP-2.

b Significant differences (P<0.05) compared to the rest of scaffolds.

c Significant differences (P<0.05) between all groups.

**Figure 3:**
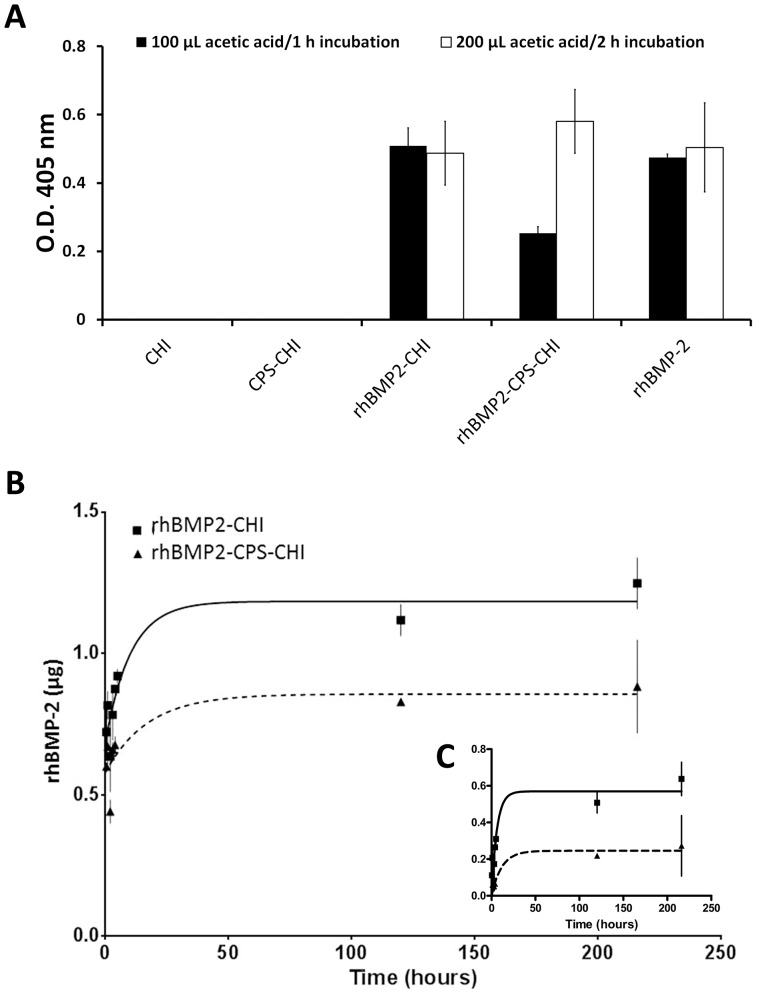
Protein activity and release. A) Graphic shows alkaline phosphatase activity induced in C2C12 cell line. This activity is induced by the active rhBMP-2 which was released from CHI scaffolds after dissolving them in 100 µL of acetic acid 50 mM for 1 hour (black columns) or 200 µL for 2 hours (white columns). B) Total rhBMP-2 detected in culture media in a time course using ELISA assay. C) rhBMP-2 release kinetic using transformed data. All data are adjusted to a first order kinetic.

At this stage, we were interested in studying whether the immobilized rhBMP-2 remained active in both rhBMP2-CHI and rhBMP2-CPS-CHI scaffolds. For this purpose, the scaffolds were dissolved in acetic acid and the osteoinductive activity of rhBMP-2 was evaluated in the resulting solution. CHI scaffolds similarly dissolved were used as the negative control and an equivalent amount of pristine rhBMP-2 was used as the positive control. Figure 3A shows that the activity of rhBMP-2 immobilized in CHI remained basically intact as compared to pristine rhBMP-2 whereas the activity of that immobilized in CHI besides CPS experienced a decrease of nearly 50%. Taking into account that the scaffold's manufacturing process was identical in both cases, and in order to explain these results, we consider the fact that the acetic acid treatment was not capable to fully dissolve the CPS of samples. It is worth noting that chromatographic columns composed of CPS (e.g. hydroxyapatite) are widely used for BMP-2 separation purposes due to the ability of CPS to adsorb BMP-2 protein [33]. Also we previously reported that rhBMP-2 is adsorbed by ceramic scaffolds [12]. Therefore, one could ascribe this data to an eventual adsorption of rhBMP-2 on non-dissolved CPS. To confirm this hypothesis, we doubled the amount of acetic acid added to dissolve the scaffold (200 µL) as well as the incubation time (2 h). After performing the alkaline phosphatase assay we observed that now all the rhBMP-2 immobilized in the rhBMP2-CPS-CHI scaffold showed nearly the same activity than rhBMP2-CHI scaffold and the positive control (Figure 3A). These results indicate that all the immobilized protein remained active after the scaffold manufacturing.

To deepen on it we were interested in studying the release of the immobilized rhBMP-2 from both rhBMP2-CHI and rhBMP2-CPS-CHI scaffolds. Figures 3B and 3C shows the release profiles found for these scaffolds. The concentration of released rhBMP-2 was determined using a sandwich ELISA kit. Raw data were initially adjusted to a first order release kinetic. Nevertheless, due to the rhBMP-2 burst liberation – nearly 50% of total amount – at very short times the initial fit was not good (see in Figure 3B the values obtained at t = 0, where data are fitted to a one phase exponential release without the restriction of Y = 0 at t = 0). Thus, we analysed the data subtracting to all of them the amount of protein released at t = 0 obtained from the previous fit. We called them transformed data which fitted then quite acceptably to a standard first order release kinetic (Fig. 3C). Comparison of fitted parameters was carried out using the extra sum-of-squares F test.

The profile shapes did not differ apparently between both scaffolds fitting to a first order release kinetic but the total amount of rhBMP-2 released from rhBMP2-CHI scaffolds was nearly 1.5-fold that released from rhBMP2-CPS-CHI scaffolds (Figure 3B). Also the rate constant (K) obtained from the fit after transforming the data differed between both scaffolds (P<0.01, Table 2, Figure 3C), indicating that there are different release kinetic curves for each data set and consequently a slower release from the rhBMP2-CPS-CHI. In both cases, the amount of rhBMP-2 released was below the amount of protein originally immobilized (8 µg). This result may be explained taking into consideration the behavior of BMP-2 immobilized in CHI. It is known that CHI swells in contact to solution (PBS or culture medium) [34]. At first, a burst liberation effect is observed and then this swelling facilitates BMP-2 diffusion to the solution. However at seven days only the 15–20% is released to the media [34]. Since there is neither acidic nor lysozime degradation in the release assay the scaffold remains almost intact in the culture medium. Thus a remarkable percentage of protein would be still immobilized in it. Finally, the differences in total released rhBMP-2 and release kinetics between these scaffolds could be due to the capability of CPS to adsorb rhBMP2 so that part of the protein would be retained – and hence, not released – within the scaffolds structure.

**Table 2:** Release kinetics parameters for rhBMP2-CHI and rhBMP2-CPS-CHI scaffolds using Y = Ymax*(1-exp-kt) after transforming raw data.

Scaffold	Y_max_	K (h^−1^)	t_1/2_	R^2^
rhBMP2-CHI	0.57±0.05**	0.16±0.04**	4.22	0.76
rhBMP2-CPS-CHI	0.22±0.04	0.09±0.07	8.01	0.61

K = rate constant expressed in hours^−1^.

** P<0.01.

**Figure 4:**
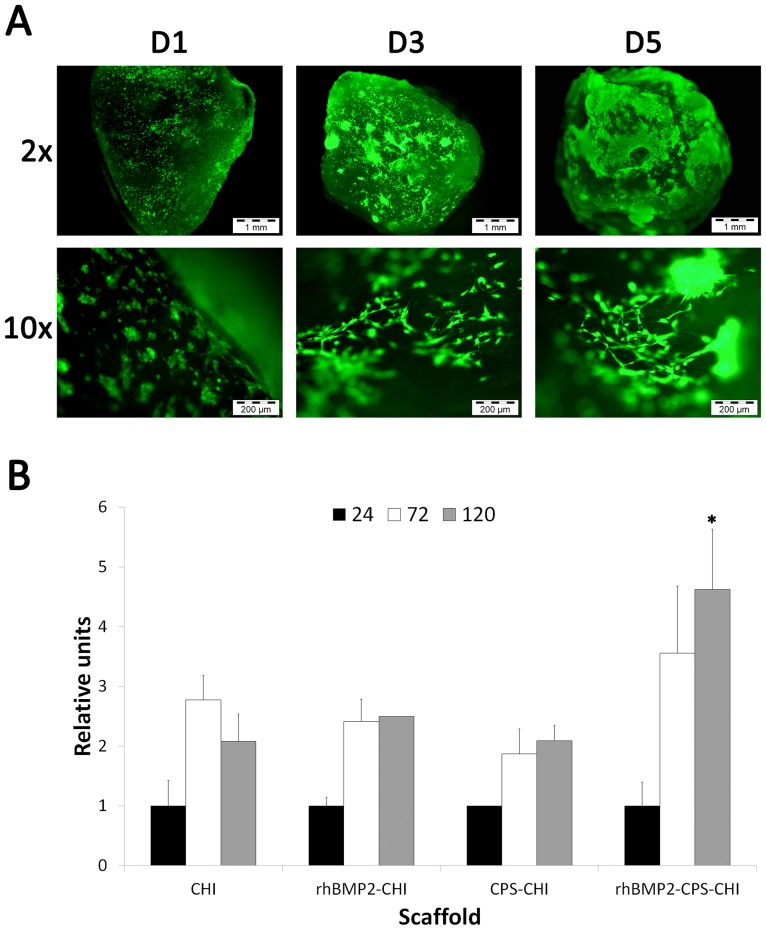
Viability and proliferation assays. A) Calcein assay: Green fluorescence corresponds to calcein vital staining at day 1 (D1), day 3 (D3) and day 5 (D5). Images show a representative experiment on rhBMP2-CPS-CHI scaffolds; B) Alamar Blue assay: Graph shows a time course quantitative measurement of cell viability for the four scaffolds (CHI, rhBMP2-CHI, CPS-CHI, rhBMP2-CPS-CHI). Relative units are referred to fluorescence units observed at day 1 in order to remark different cell growth between different scaffolds. *P<0.05 comparing with the other scaffolds at the same time point.

We next studied cell cultures on scaffolds of 4.5 mm in diameter and ca. 2.5 mm in height that were obtained by simple cryo-fracture of the original monoliths obtained from ISISA. Cultured cells were stained with calcein that labels live cells with bright-green fluorescence. This assay, performed on rhBMP2-CHI and rhBMP2-CPS-CHI scaffolds, was extremely useful to observe (1) the existence of cells attached to the scaffolds (2) how cells morphology evolved during the culture – e.g. from well-rounded into fibroblastic-type – within the following days (Figure 4A). After this first qualitatively assessment, the Alamar Blue assay was performed to quantify cell proliferation (Figure 4B). Cells proliferate on CHI, rhBMP2-CHI and CPS-CHI scaffolds over the first three days. Cell proliferation was slightly higher on rhBMP2-CPS-CHI scaffolds at this time-point and further increased – with a significant statistical difference (P<0.05) – over the following two days (e.g. after 5 days). Neither CPS-CHI nor rhBMP2-CHI scaffolds exhibited this proliferation trend. In these two cases, proliferation figures were just maintained after three days of culture whereas in CHI scaffolds experienced a slight decrease. Thus, data suggest a synergistic effect of substrate properties – CPS incorporation and growth factor activity – on cell proliferation rates.

**Figure 5:**
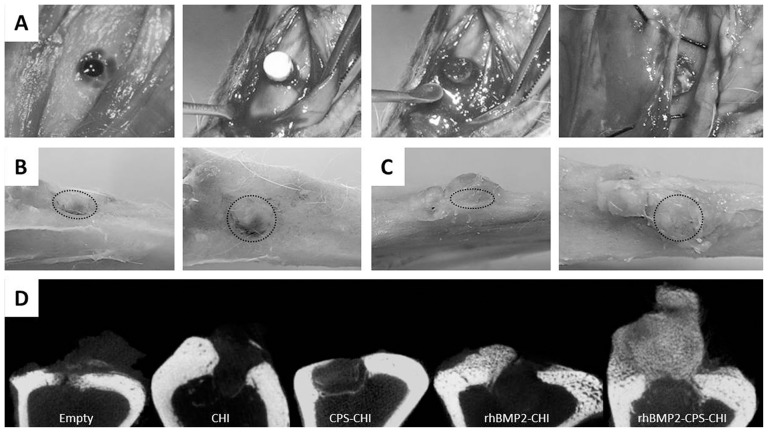
Surgery (A), gross morphology after euthanasia (B, C) and microCT analysis of samples (D). Surgery images (A) show scaffold implantation process. After euthanasia gross morphology images were obtained at different angles (B,C) (Dotted circle indicates defect location). Defect area is still observed in CPS-CHI implanted tibias (B), while in rhBMP2-CPS-CHI implanted tibias high amount of newly-formed hard tissue, apparently bone, appeared vertically from the defect (C). MicroCT study (D) confirms trabecular bone formation in rhBMP2-CPS-CHI implanted tibias, while it seems no scaffold-resorption in any case. Neither seems a robust new bone formation in the rest of implanted scaffolds compared to empty controls.

Then we proceeded to evaluate the *in vivo* osteoinductive response of the scaffolds. For this purpose and as described in experimental part, 4 mm-in-diameter defects were created in rabbit tibias. The formation of bone was studied by allocating every single scaffold in its respective defect over three weeks, using an empty defect – without any scaffold – as control. The scaffolds were all of identical size and weight (e.g. 4 mm in diameter, 2.5 mm in height), so that they fitted well into the defects. After three weeks of implantation, microCT and histological studies were performed. Figure 5A shows the surgery process and the tibia gross morphology after euthanasia (B,C). The defects filled with rhBMP2-CPS-CHI scaffolds exhibited the formation of a major fraction of dense bone-resembling new tissue at the periostium whereas only a minor fraction of soft tissue was observed in the defects filled with CPS-CHI scaffolds. MicroCT analysis (Figure 5D and S1) confirmed the formation of trabecular bone in the former case, but neither the latter nor the defects filled with CHI or CPS-CHI scaffolds followed this trend. Surprisingly, rhBMP2-CHI apparently shows no more new bone formation compared to CHI implanted samples, suggesting a role of CPS in rhBMP-2 induced bone formation. Being a critical feature for bone formation the proper release of BMP-2 from the scaffold [16], [17], it seems that rhBMP-2-CHI scaffold do not provide an effective delivery frame of included rhBMP-2 in order to allow bone formation.

**Figure 6:**
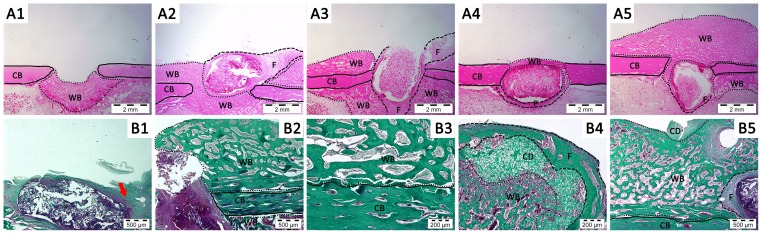
Histology. A) Hematoxylin-eosin staining. A1) Empty control, A2) CHI scaffold, A3) rhBMP2-CHI scaffold, A4) CPS-CHI scaffold, A5) rhBMP2-CPS-CHI scaffold. Different lines are limiting newly formed woven bone (WB, **···**) with resembles trabecular bone structure (intramedular as well as extramedullar), cortical bone (CB, **—**) and fibrous tissue (F, **– – –**). All images were taken at 2×. B) Masson's Trichrome staining. B1) CPS-CHI scaffold. A little amount of bone is formed, and a connective tissue (red arrow) is observed around the matrix, B2-5) rhBMP2-CPS-CHI scaffold. Great amount of new bone is formed, most of it extramedullar trabecular bone (WB). Detailed image (B3) shows a dotted line limiting native cortical bone (CB, —) and newly formed woven bone (WB). Chondral areas (CD) appear in the limits of newly formed tissue (B4, B5) here limited with woven bone indicating new bone formation via osteochondral ossification. Images B1, B2 and B5 were taken at 5×. Images B3 and B4 were taken at 10×. All histological slides were from the closest zone to the center of the defect as possible.

Histological analysis was carried out using hematoxylin/eosin and Masson's trichrome staining. Hematoxylin/eosin images in figure 6 show the formation of collagen-rich connective tissue in every case, both in the empty defect as well as in the defects filled with the different scaffolds. Tissue formation in the empty defect mainly occurred at the intramedular space. Bone healing process has been previously explained in this work [7]. Therefore, this feature was not surprising given the intrinsic capacity of bone for self-repair and/or self-regenerate in response to a fracture when this is below a certain critical size. In fact similar bone formation is described in clinically performed corticotomies and distraction osteogenesis surgeries [35]. Actually, in all tested cases newly formed woven bone was observed, because of native bone regeneration process.

With regard to the implanted scaffolds, the histological study (Figure 6) revealed that none scaffold was degraded after three weeks of implantation and they remained in the defect area. In every case, the first obvious observation was the formation of a fibrous capsule at the implant-tissue interface, the thickness of which around the different scaffolds did not show noticeable differences (Figure 6). Fibrous capsule formation is a well-established reaction to implanted biomaterials and is recognized as the end stage of the foreign body reaction [36]. Neither the scaffolds pores nor the inner interstice between the scaffolds and the fibrous capsule exhibited a remarkable formation of new tissue. Actually, new tissue was only observed around the scaffolds. Particularly noticeable was the formation of tissue in rhBMP2-CPS-CHI scaffold, the morphological features of which resembled those of trabecular bone (Figure 6). In this case, we used the Masson's trichrome stain, the broad use of which in bone tissue studies comes from its specific capability to stain collagen, hence allowing better contrast between newly formed tissues as compared to hematoxylin/eosin stains. Interestingly, the presence of chondral zones adjacent to woven bone revealed that endochondral ossification – typically observed in naturally-occurring bone healing processes – also played a role in the rhBMP2-induced process of bone regeneration described in this work. Note that histology revealed once again no additional bone formation in rhBMP-2-CHI samples.

**Figure 7:**
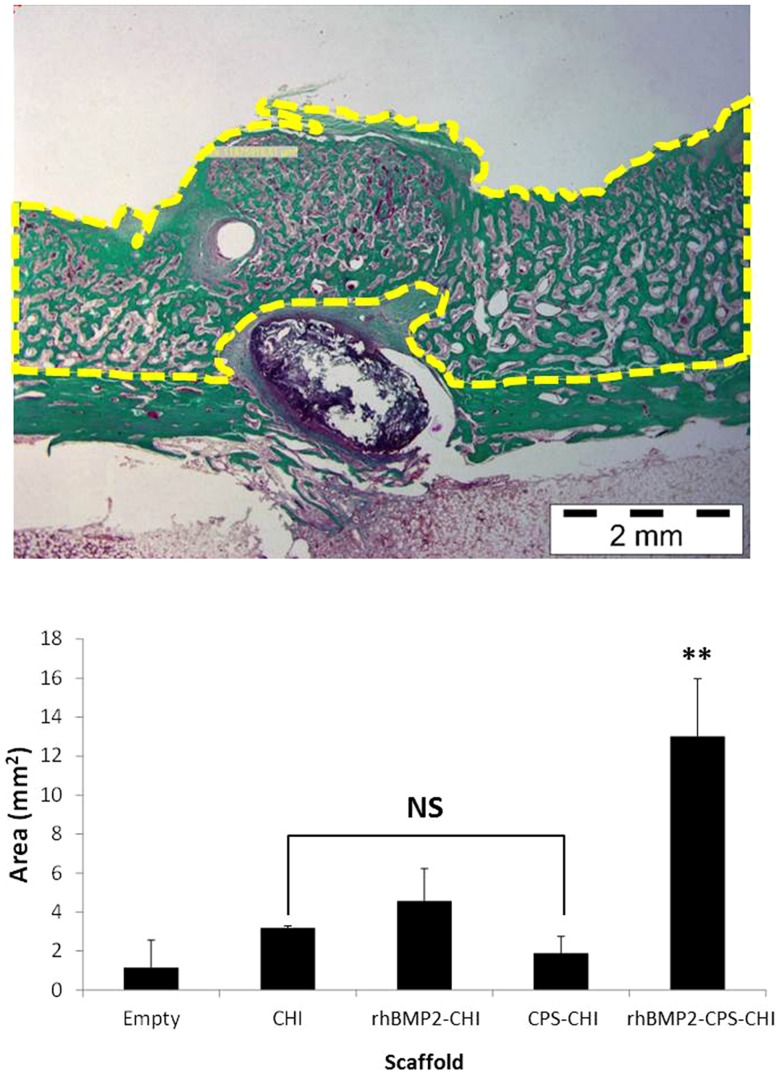
Histomorphometry. Image is shown as an example of measurements performed and corresponds to a rhBMP2-CPS-CHI sample. Histogram shows the mean area values obtained in each treatment for newly-formed bone (NS: statistically not significant compared to control, **P<0.01).

We decided to evaluate the capability of bone regeneration eventually induced by the presence of the scaffolds by considering the amount of new tissue that was formed in every case. For this purpose, we applied histomorphometric analysis to the slides stained with Masson's trichrome (Figure 7). The statistical analysis showed similar newly-formed bone amount for empty controls and CHI, CPS-CHI and rhBMP2-CHI scaffolds. Nevertheless, rhBMP2-CPS-CHI scaffolds formed significantly higher amount of new bone (P<0.01). This result revealed that the efficacy in bone regeneration of the scaffolds studied in this work was superior when mineralization agents (e.g. CPS) and bone morphogenetic proteins (e.g. rhBMP-2) act in a combined fashion rather than separately.

## Conclusions

We have prepared CHI scaffolds containing both CPS and rhBMP2. We have observed that rhBMP2 was not only released in a controlled fashion from CHI scaffolds but also preserved its osteoinductive character after release. Interestingly, we found that this multicomponent scaffold exhibited a superior efficacy in bone regeneration than the scaffolds containing only one of the components, either CPS or rhBMP2, separately. This enhanced performance in both osteoconductive and osteoinductive terms opens the path to the future clinical application of these materials in dental surgery and, more specifically, in maxillary sinus augmentation procedure. In this procedure, large area of the maxillary sinus are lifted and replaced with bone, which serves to support future implant placement. It is worth noting that the filling material most used nowadays is porous resorbable hydroxyapatite, which is osteoconductive but not osteoinductive as the rhBMP2-CPS-CHI scaffolds described in this work.

## Supporting Information

Figure S1 **MicroCT images of rhBMP-2-CPS-CHI scaffolds implanted in rabbit tibias.** Trabecular bone formation is observed in all cases.
(TIF)
