# Supplementary figures and images for: Chitosan Scaffolds Containing Calcium Phosphate Salts and rhBMP-2: In Vitro and In Vivo Testing for Bone Tissue Regeneration
Source: PLoS One. 2014 Feb 4;9(2):e87149. doi: 10.1371/journal.pone.0087149 (PMC3913585; doi:10.1371/journal.pone.0087149)

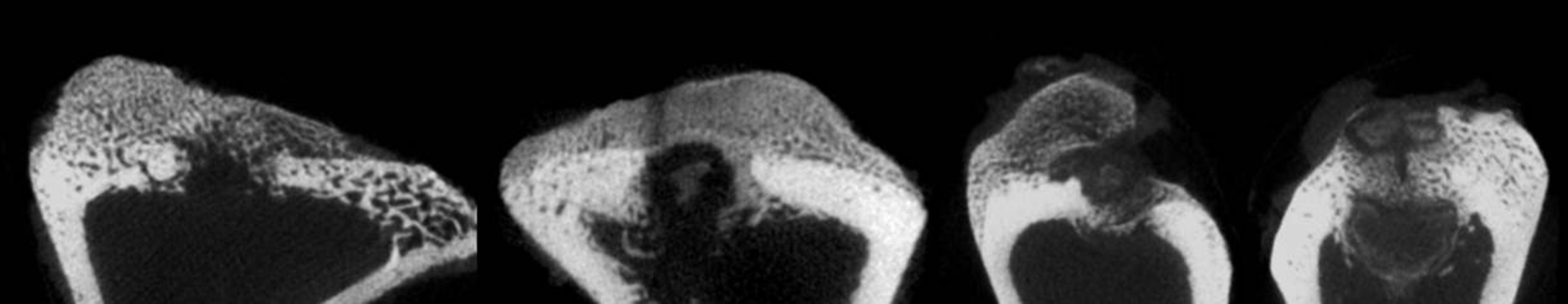

Supplement: Figure S1 — MicroCT images of rhBMP-2-CPS-CHI scaffolds implanted in rabbit tibias. Trabecular bone formation is observed in all cases. (TIF) [file pone.0087149.s001.tif]
